# Editorial: Insights in emerging or re-emerging zoonoses

**DOI:** 10.3389/fvets.2024.1454535

**Published:** 2024-07-16

**Authors:** Javier Caballero Gómez, Santiago Mirazo, Mustafa Altindis, Barbara Moroni

**Affiliations:** ^1^Center for Biomedical Research in Infectious Diseases, Carlos III Health Institute (ISCIII), Madrid, Spain; ^2^Clinical Virology and Zoonoses Group, Maimonides Biomedical Research Institute of Cordoba (IMIBIC), Córdoba, Spain; ^3^Animal Health and Zoonoses Research Group, University of Cordoba, Córdoba, Spain; ^4^Departamento de Bacteriología y Virología, Instituto de Higiene, Facultad de Medicina, Universidad de la República, Montevideo, Uruguay; ^5^Department of Medical Microbiology, Faculty of Medicine, Sakarya University, Sakarya, Serdivan, Türkiye; ^6^Experimental Zooprophylactic Institute for Piedmont, Liguria and Valle d'Aosta (IZSPLVA), Turin, Italy

**Keywords:** public health, zoonoses, emerging, epidemiology, surveillance

The recent COVID-19 pandemic and the frequent cross-species transmission from birds to mammals of highly pathogenic avian influenza (HPAI) A(H5N1) strikingly underscore that the battle against emerging infectious diseases is far from over.

According to the World Health Organization (WHO), 60% of human pathogens originate from animals, and 75% of pathogens responsible for emerging and reemerging animal diseases have the potential to cross the animal-human interface. Thus, the emergence of zoonotic diseases is a global multifaceted problem that should be addressed through an integrated approach by the scientific community.

The main goal of this Research Topic was to provide a comprehensive collection of research studies on the epidemiology, diagnosis and pathology of infections caused by emerging or re-emerging zoonotic pathogens, including viruses, parasites, and bacteria affecting humans, domestic animals and wildlife. The 13 manuscripts of this Research Topic explore different aspects of emerging infectious diseases across the globe, including 10 original research studies, one review, and two brief research reports.

As a major outcome, around 70% of the published papers are related to viral pathogens, with a special focus on Asian countries, while the remaining focus on bacterial and parasitic diseases across the globe. Both domestic animals (companion and livestock) and wildlife have been investigated throughout the Research Topic as a potential source of infectious diseases with zoonotic potential in different socioeconomical and environmental contexts.

A strong link between poor animal welfare levels and zoonotic risks has been found by Mace and Knight in their review describing risk factors for the transmission of avian influenza (AI). They highlighted how the risks from industrial animal farming, especially mixed swine and poultry farms, are high. Reducing stress in farmed animals is crucial for their immunocompetence and to minimize disease risks. The authors advise against intensive farms and recommend lowering stocking densities to improve animal health and welfare.

Hinjoy et al., explored how poultry movement patterns in Thailand may explain AI spread over the networks once introduced using network analysis approaches. In 2021, poultry trades were recorded among 338 farmers and eight traders. The authors mapped 99 subdistricts and 181 trade links, finding feedback loops in 56 subdistricts. This network illustrates that the poultry trade in three border provinces of Thailand was relatively localized highlighting potential AI spread pathways. Enhanced surveillance and control in high-trade areas can mitigate AI transmission, and social network analysis improves risk communication and biosecurity, enabling targeted strategies for more effective and efficient disease risk reduction across the value chain.

The evolution and possible origin of influenza viruses isolated from bats and birds is reported by Karamendin et al.. Bat H9 viruses likely originated from avian H9, with neutralizing antibodies detected in African bats. The Caspian region, a major migratory route, could facilitate viral mixing in different species. Increasing human H9N2 infections in Asia raise pandemic concerns, highlighting the need for regular monitoring of H9 virus in animal and human populations to ensure pandemic preparedness.

Different international organizations, such as WHO or EFSA, have pointed out the need to monitor other emerging and zoonotic viruses, such as SARS-CoV-2. In this Research Topic, a large-scale survey was carried out in Thailand by describing the sero-epidemiology of this virus in dogs and cats during the first and fourth waves of the Thailand COVID-19 outbreak between 2020 and 2021. The results of their study revealed seropositivity in eight out of 3,099 individuals sampled (0.26%), specifically in three cats and five dogs. Ongoing surveillance in companion animals, especially cats, is crucial due to their potential susceptibility to new variants. This could create viral reservoirs and facilitate human-animal transmission.

It is of paramount importance to maintain active surveillance of zoonotic emerging infectious diseases also at the livestock/human interface and wildlife/human interface, as highlighted by Ryoo et al. and Zhou et al., they report infection with a novel strain of foot and mouth disease virus (FMDV) in cattle and goat and Parainfluenza virus 5 (PIV5) in a 12-year-old captive male Siberian tiger from China, respectively. While Ryoo et al. provided evidenced how emergency vaccination and intensive surveillance programs reduced the spread of the new FMDV strain, Zhou et al. underlines the urgent need to control PIV5 in zoo animals to prevent interspecies transmission. PIV5 mutations in wild animals offer potential candidates for researching virus evolution and transmission mechanisms.

As a re-emerging neglected zoonosis, rabies represents one of the most significant public health concerns worldwide due to its fatal evolution. The study by Punyapornwithaya et al. aimed to predicting the incidence of canine rabies in Thailand using time series methodologies. They identified 4,678 confirmed canine rabies cases, showing seasonal patterns. The TBATS model demonstrated good predictive accuracy, forecasting an annual average of 285 cases (23 monthly) for 2023–2025. The study by Punyapornwithaya et al. offers advanced time series methodologies for infectious disease forecasting, introducing surveillance methods for rabies prevention, and control efforts.

Another comprehensive contribution on rabies has been done by Cunha et al., who reported the circulation of three Lyssavirus variants and their epidemiological importance in the north and northeast regions of Brazil, which harbor a large diversity of hosts, including bats. As highlighted by the authors, the emergence of Lyssavirus, facilitated by diverse bats and interspecies interaction, poses risks of spillover events and new viral strains. Marmosets (*Callithrix jacchus*) in Brazil serve as a unique reservoir for rabies, with implications for human infections. Control efforts should focus also on urban rabies while major challenges persist with wild animals.

Bacterial infections can also be causative agents of important zoonotic emerging or re-emerging diseases, such as brucellosis and salmonellosis. In their cross-sectional study, Ahad et al. described seroprevalence of brucellosis in camels, sheep, and goats, and their owners from Ethiopia, and analyzed risk factors collected via questionnaire. The study revealed a 5% brucellosis seroprevalence in camels, sheep, goats, and humans. Herd size and retained fetal membranes were risk factors for animal seropositivity, while human seropositivity correlated with contact with seropositive animals and assisting during calving or birthing. This study highlights again the usefulness of the “One Health” approach while planning control strategies and surveillance programs.

Even in remote islands such as the Cayman Islands (Caribbean), the epidemiological surveillance of zoonosis at the wildlife/livestock interface is of paramount importance to prevent new outbreaks in humans. Watler et al., investigated whether Salmonella could be introduced in the local poultry by import through the collection of boot swabs, paper bedding, and cecum samples from imported and feral chickens in the Cayman Islands. The results highlighted that Salmonella in the Cayman Islands mainly originates from imported day-old chicks. While feral chicken serotypes match those commonly found in humans, their low virulence and the absence of antimicrobial resistance mitigate overall health risks. Antibiotic resistance is concerning and underscores the need for stricter import controls and ongoing surveillance programs.

In particular, multi-drug resistance (MDR) has been considered a major plague globally in recent years, and both livestock and companion animals are considered sentinels to prevent this phenomenon in humans. Abad-Fau et al., reported an alarmingly high percentage (71.15%) of multidrug resistant *E. coli* from urine samples of dogs suffering from urinary infections in Spain, underlying how dogs may harbor MDR pathogenic *E. coli*, potentially facilitating antimicrobial resistance transmission to humans. Continuous surveillance of antimicrobial resistance and updating therapeutic guidelines is imperative in veterinary clinics to mitigate these concerns.

While of minor spread, *Streptococcus suis* can be an important zoonotic pathogen, especially if considered as an occupational disease in the swine sector causing a wide range of fatal diseases (among others, septicemia, endocarditis, meningitis, and pneumonia) in humans. Zong et al., investigated genes and metabolites crucial for the survival of *S. suis* 2 aiming to identify potential targets for preventing and managing *S. suis* 2 infections.

Finally, some parasitosis can be regarded as significant re-emerging neglected zoonotic diseases, such as leishmaniosis caused by *Leishmania infantum*. Domestic dogs are the most important reservoir of this parasitic protozoa, and surveillance to enable early diagnosis is key to controlling it. In this regard, Sarquis et al. contributed with novel important insights in the management of canine leishmaniosis through the validation of a new clinical diagnostic biomarker. Circulating immune complexes were tested as potential biomarkers to diagnose canine leishmaniosis, and also to track the progression of the disease. Again, from a One Health perspective, this study will further contribute to prevent and control a major parasitic disease that can affect humans and animals.

## Author contributions

JC: Writing – original draft, Writing – review & editing. SM: Writing – original draft, Writing – review & editing. MA: Writing – original draft, Writing – review & editing. BM: Writing – original draft, Writing – review & editing.

